# Does D3 surgery offer a better survival outcome compared to D1 surgery for gastric cancer? A result based on a hospital population of two decades as taking D2 surgery for reference

**DOI:** 10.1186/1471-2407-10-308

**Published:** 2010-06-20

**Authors:** Hao Zhang, Caigang Liu, Di Wu, Yi Meng, Ruonan Song, Ping Lu, Shubao Wang

**Affiliations:** 1 Department of Surgery Oncology, General Surgery, First Hospital of China Medical University, Shenyang, China; 2 Department of Cardiology, First Hospital of China Medical University, Shenyang, China; 3 Department of Orthopaedics, First Hospital of China Medical University, Shenyang, China; 4 Department of Anesthesiology, First Hospital of China Medical University, Shenyang, China

## Abstract

**Background:**

We conducted a retrospective study in our hospital in which we compared D1 with D3 through D2 lymphadenectomy for gastric cancer in terms of morbidity, postoperative mortality, long-term survival after surgery.

**Methods:**

567 patients who were performed curative intent between 1980 and 2003 were enrolled. 187 in the D1 group, 189 in the D2 group and 191 in the D3 group. Every procedure was verified by pathological analyses. The primary endpoints were 5-year overall survival.

**Results:**

Median follow-up periods were 36 months and 60 months for D1 group and D3 group. Overall 5-year survival rate was significantly higher in patients underwent D3 surgery than in those performed D1 surgery (37.4% vs 48.7%; log-rank, p = 0.027). For the cases followed up to 120 months, the 10-year overall survival rate was 29% (95% CI, 22.1% to 35.9%) for the D1 group and 33.7% (95% CI, 26.6% to 40.8%) for the D3 group (log-rank, p = 0.005).

**Conclusions:**

D1 surgery should be operated only for patients with Borrmann I disease. As D3 gastrectomy is associated with low mortality and adequate survival times when performed in selected institutions that have had sufficient experience with the operation and with postoperative management, we recommend D3 lymphadenectomy for patients with curable gastric cancer.

## Background

Gastric cancer is still the most common cause of cancer related deaths worldwide, and a major clinical problem needing to be resolved because of the poor prognosis and the leak of treatment methods. Nowadays, surgical management is the major treatment method for gastric cancer. However, the efficacy of various extent of nodal dissection is still under debate. It was reported that improved prognosis was got in patients with gastric cancer who underwent D3 lymphadenectomy (first edition of Japanese classification of gastric cancer[1-3]. Furthermore, there were some randomised multi-institutional trials showing no survival benefits, but high morbidity and mortality, after D3 gastric dissection compared with D1 dissection[4,5]. To be mentioned, there were many participating surgeons with little experience in D3 surgery in these trials, hence, it's difficult to control the quality[4,5].

The more extended the surgery, the greater the risk of operation related morbidity and mortality is, as reported previously that nodal dissection increased morbidity[6]. It was reported that the postoperative mortality rate for gastrectomy surgery often exceeds 5% in West, even gets close to 16% in some articles,[7-9] only some Japanese studies reported a lower than 2%[10]. Besides the operation related morbidity, there was also a report showing that lymph node dissection did not adversely influence QOL,[11] and the operation related morbidity did not influence survival[12,13].

We conducted a single-institutional study and reported the long-term survival data for these two surgical groups of D1 and D3 taking D2 group as reference. Finally, we demonstrated that D3 surgery has overall survival benefit without significant operative complications and mortality.

## Methods

### Patients

We selected 567 patients who were histologically confirmed gastric cancer and underwent a radical operation at the First Affiliated Hospital of the China Medical University between 1980 and 2005. All of them, 187 were performed D1 dissection (D1 group), 189 received D2 surgery (D2 group) and 191 were treated with D3 dissection (D3 group). The inclusion criteria were as follows: 1), histologically proven, potentially curablengastric adenocarcinoma, and had physical fitness suitable for elective operation of either type of lymphadenectomy; 2), diagnosed based on the 5th UICC TNM classification system; 3), curative D1, D2 or D3 operations were performed; 4), a complete medical record was available; 5), patients of every period of diagnosis and every surgeon are roughly equal; and 6), never received neoadjunctive therapies and any kind of adjunctive therapy. Exclusion criteria were as follows: 1), older than 75 years; 2), previous or concomitant other cancer; 3), previous or concomitant gastrectomy for benign disease; 4), previous chemotherapy or radiotherapy; 5), clinical evidence of early gastric cancer on laparotomy; 6), oesophageal involvement; 7), macroscopically enlarged lymph nodes around the hepatoduodenal ligament or para-aortic regions; and 8), distant metastatic disease.

All patients were followed up by posting letters or telephone interviews. The last follow-up was December, 2008. Clinical findings, surgical findings, pathological findings and every follow-up were collected and recorded in the database. All the subjects gave written informed consent to study protocol, which was approved by the Ethics Committee of China Medical University.

### Surgical procedures and classifications of gastric cancer

Surgical procedures and pathological assessment refered to the Japanese classification of gastric cancer[1]. All patients in the study underwent standard total or distal subtotal gastrectomy, depending on the location and macroscopic appearance of the primary tumor. The definition of lymphadenectomy was based on the Japanese Classification of Gastric Carcinoma[5]. D1--dissection of all the group 1 nodes; D2--dissection of all the group 1 and group 2 nodes; D3--dissection of all the group 1, group 2 and group 3 nodes. Group 1 consists of the perigastric lymph nodes, and group 2 consists of the lymph nodes along the left gastric artery, the common hepatic artery, and the splenic artery and around the celiac axis. However, when the tumor is located in the lower third stomach, the lymph nodes along the splenic artery are classified as group 3. Group 3 also consists of lymph nodes in the hepatoduodenal ligament at the posterior aspect of the head of the pancreas and at the root of the mesentery.

Surgeons routinely removed lymph nodes from the excised specimens as more as possible after operation, based on the Japanese Classification of Gastric Carcinoma and their experience. The specimens and retrieved lymph nodes were stained with hematoxylin and eosin and pathologically examined in the Gastric Laboratory of the First Affiliated Hospital of the China Medical University.

### Endpoints and follow-up

The primary endpoints were 5-year overall survival. Overall survival was calculated from the day of surgery until death or the last follow-up contact. Data for a patient were censored at last follow-up when they were alive. Follow-up assessments were done every 6 months for the first 5 years after surgery, and then every 12 months until death.

### Statistical analyses

Data from all eligible patients were analyzed for overall survival. Survival curves were estimated by the Kaplan-Meier method and treatment comparisons were made by the log-rank test.

Potential prognostic factors were entered into a Cox's regression model including age, sex, tumor size, site of tumour in stomach, gross appearance, tumour stage, clinical node status, nodal stage, joint organ removal, gastrectomy, blood transfusion and blood loss. In multivariate analysis, the prognostic factor detected in univariate analysis and treatment group were as covariates included in the Cox regression model.

Two-sided P values were calculated for all tests and are reported here. P values less than 0.05 were considered to indicate statistical significance. Analyses were performed with the use of SPSS software, version 16.0.

## Results

D1 group was with median age of 55 years old, D2 group was with median age of 55 years old and D3 group was with median age of 54 (table 1). All patients were followed up for at least 5 years (until December 19, 2008).

**Table 1:** Characteristics of D1, D2, D3 population (n = 567)

Characteristics	D1 surgery (n = 187)	D2 surgery (n = 189)	D3 surgery (n = 191)	p Value
**Age (years)**				0.899
Median	55	55	54	
**Sex**(%)				0.758
Men	136(73)	137(73)	133(70)	
Women	51(27)	52(27)	58(30)	
**Number of lymph nodes removed**				0.189
Mean	21	23	26	
**Number of involved lymph nodes**				0.232
Mean	3	4	6	
**Tumor size**(%)				0.323
≤5 cm	125(67)	121(64)	113(59)	
5-7 cm	32(17)	40(21)	50(26)	
>7 cm	30(16)	28(15)	28(15)	
**Site of tumour**(%)				0.984
Upper stomach	44(24)	39(21)	42(22)	
Middle stomach	43(23)	50(27)	45(24)	
Lower stomach	88(47)	87(46)	90(47)	
Whole stomach	12(6)	13(6)	14(7)	
**Pathological tumour stage**(%)				0.979
T1	41(22)	40(21)	44(23)	
T2	44(24)	45(24)	42(22)	
T3	89(48)	87(46)	92(48)	
T4	13(6)	17(9)	13(7)	
**Clinical node status**(%)				0.729
Positive	182(97)	186(98)	186(97)	
Negative	5(3)	3(2)	5(3)	
**Pathological nodal stage**(%)*				
N0	69(37)	60(32)	69(36)	0.500
N1	75(40)	93(49)	81(42)	
N2	30(16)	26(14)	24(13)	
N3	13(7)	10(5)	17(9)	
**Gross type**(%)				0.998
Borrmann I	58(31)	56(30)	54(28)	
Borrmann II	41(22)	44(23)	43(23)	
Borrmann III	73(39)	74(39)	77(40)	
Borrmann IV	15(8)	15(8)	17(9)	
**Histological type**(%)				0.867
Differentiated	99(53)	88(47)	82(43)	
Undifferentiated	88(47)	101(53)	109(57)	
**Curative resection**(%)	174(93)	181(96)	181(95)	0.503
**Type of gastrectomy**(%)				0.440
Total	22(12)	21(11)	29(15)	
Subtotal	165(88)	168(89)	162(85)	
**Combined organ resection**				0.283
Pancreas or spleen	7(4)	16(9)	14(7)	
Liver or gall	11(6)	8(4)	9(5)	
Transverse colon	9(5)	17(9)	11(6)	
**Blood transfusion**(%)	100(54)	104(55)	111(58)	0.652

*N1 = 1-6 involved nodes; N2 = 7-15 involved nodes; N3, >15 involved nodes.

The characteristics of the three groups, which were showed in table 1, were well balanced. 125 patients had early cancer (confined to submucosa or mucosa). 536 patients had curative resection; 31 patients had palliative resection. 22 (12%) patients in D1 group was performed total gastrectomy, 21 patients assigned to D2 group (11%) and in 29 patients assigned to D3 surgery (15%). The incidence rates of the four major surgery-related complications in the D1 group, D2 group and D3 group were 2% (4/187), 2% (4/189) and 2% (3/191), respectively, for anastomotic leakage; 4% (8/187), 4% (7/189) and 5% (9/191) for pancreatic fistula; 4% (8/187), 5% (10/189) and 5% (10/191) for abdominal abscess, and 4% (7/187), 2% (3/189) and 1% (2/191) for pneumonia. None of these differences were statistically significant (all P > 0.05). The hospital death rate was 2% (three deaths in D1 group, one death in D2 group and six deaths in D3 group).

**Figure 1:**
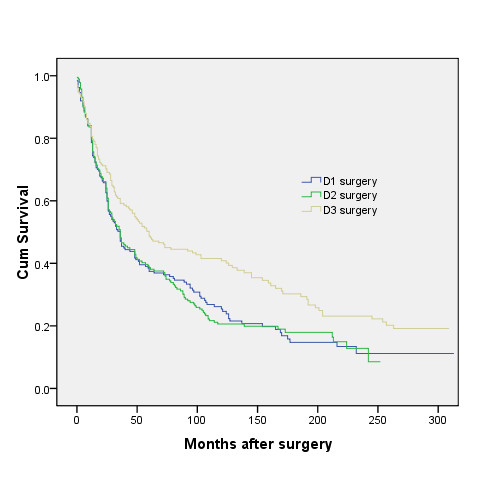
**The overall rates for all enrolled patients**. There were significant differences between D1 and D3 group (p = 0.004), and between D2 and D3 group (p = 0.002). However, there was no significant difference between D1 and D2 group.

**Figure 2:**
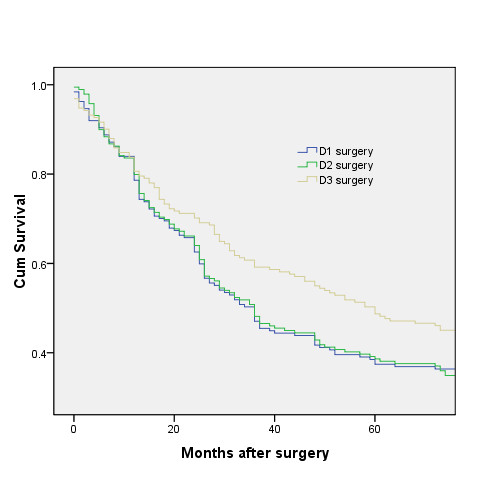
**The 5-year overall survival for all enrolled patients**. The 5-year overall survival was 37.4% (95% CI, 30.5% to 44.3%) for the D1 group and 48.7% (95% CI, 41.6% to 55.8%) for the D3 group (log-rank p = 0.027).

After median follow-up periods of 36 months, 36 months and 60 months for D1 group, D2 group and D3 group respectively, 150 patients in D1 group, 157 patients in D2 group and 137 in D3 group died. Neither the skill of an individual surgeon nor the period of diagnosis affected survival (p > 0.05, log-rank test). Figure 1 and figure 2 show the overall rates for all enrolled patients. There were significant differences between D1 and D3 group (p = 0.004), and between D2 and D3 group (p = 0.002). However, there was no significant difference between D1 and D2 group. The 5-year overall survival was 37.4% (95% CI, 30.5% to 44.3%) for the D1 group and 48.7% (95% CI, 41.6% to 55.8%) for the D3 group (log-rank p = 0.027), and for those whose follow-up periods were up to 120 months, the 10-year overall survival was 29% (95% CI, 22.1% to 35.9%) for the D1 group and 33.7% (95% CI, 26.6% to 40.8%) for the D3 group (log-rank p = 0.005).

The hazard ratio for death was 0.708 (95% CI, 0.560-0.894; p = 0.004) in the D3 group (table 2, univariable analyses). After adjustment of thirteen baseline variables (age, sex, tumor size, tumor location, Borrmann type, T stage, clinical node status, lymph-node stage, histological type, joint organ removal, gastrectomy, blood transfusion and blood loss) with the use of Cox regression analysis, the hazard ratio was hardly unchanged (hazard ratio, 0.771 (95% CI, 0.599-0.992); P = 0.043) (table 2, multivariable analyses). Expectedly, the multivariate analyses showed that >7 cm in tumor size, the upper third tumor and the whole stomach tumor, Borrmann III type, N3 disease, D1 and D2 dissection were significantly associated with poor survival (table 2).

**Table 2:** HR for death in intention-to-treat population (n = 567)--univariable and multivariable analyses

	**Univariable analyses**		**Multivariable analyses**	
	
	**HR (95% CI)**	p*	**HR (95% CI)**	**p†**
**Age (years)**		**0.033**		**0.335**
≤55	1 (Ref)		1 (Ref)	
>55	1.231(1.017-1.490)	0.033	1.224(0.982-1.524)	0.335
**Sex**		**0.816**		**0.960**
Women	1 (Ref)		1 (Ref)	
Men	1.025(0.833-1.261)	0.816	0.943(0.748-1.188)	0.960
**Tumor size**		**0.000**		**0.111**
≤5 cm	1 (Ref)		1 (Ref)	
5-7 cm	1.527(1.217-1.917)	0.000	1.266(0.991-1.617)	0.059
>7 cm	1.728(1.338-2.233)	0.000	1.397(1.005-1.943)	0.047
**Tumour site**		**0.101**		**0.005**
Upper stomach	1 (Ref)		1 (Ref)	
Middle stomach	0.624(0.477-0.816)	0.001	0.585(0.436-0.786)	0.000
Lower stomach	0.626(0.495-0.792)	0.000	0.634(0.482-0.833)	0.001
Whole stomach	1.303(0.889-1.910)	0.174	0.698(0.422-1.154)	0.161
**Gross appearance**		**0.000**		**0.000**
Borrmann types I	1 (Ref)		1 (Ref)	
Borrmann types II	1.080(0.824-1.416)	0.576	0.979(0.740-1.294)	0.879
Borrmann types III	1.723(1.364-2.175)	0.000	1.601(1.253-2.046)	0.000
Borrmann types IV	2.141(1.507-3.044)	0.000	1.282(0.841-1.952)	0.248
**Tumour stage**		**0.000**		**0.651**
T1	1 (Ref)		1 (Ref)	
T2	0.855(0.638-1.146)	0.295	0.799(0.590-1.082)	0.146
T3	1.262(0.956-1.615)	0.064	0.917(0.698-1.205)	0.535
T4	1.867(1.284-2.716)	0.001	1.005(0.657-1.537)	0.983
**Clinical node status**		**0.372**		**0.438**
Negative	1 (Ref)		1 (Ref)	
Positive	1.376(0.683-2.770)	0.372	1.652(0.794-3.436)	0.438
**Lymph-node stage**		**0.000**		**0.000**
N0	1 (Ref)		1 (Ref)	
N1	1.342(1.082-1.666)	0.008	1.119(0.863-1.450)	0.397
N2	1.480(1.099-1.994)	0.010	1.238(0.889-1.725)	0.206
N3	3.603(2.497-5.199)	0.000	2.653(1.684-4.179)	0.000
**Histological type**(%)		**0.221**		**0.556**
Differentiated	1 (Ref)		1 (Ref)	
Undifferentiated	1.177(0.591-1.529)	0.221	0.897(0.624-1.289)	0.556
**Joint organ removal**		**0.000**		**0.000**
None	1 (Ref)		1 (Ref)	
Pancreas or spleen	1.964(1.374-2.808)	0.000	1.364(0.902-2.062)	0.141
Liver or gall	1.380(0.896-2.125)	0.144	1.291(0.823-2.023)	0.266
Transverse colon	1.882(1.321-2.681)	0.000	1.446(0.984-2.125)	0.060
**Gastrectomy**		**0.000**		**0.001**
Total	1 (Ref)		1 (Ref)	
Subtotal	0.549(0.422-0.714)	0.000	0.833(0.563-1.233)	0.001
**Blood transfusion**		**0.356**		**0.580**
No	1 (Ref)		1 (Ref)	
Yes	1.094(0.904-1.324)	0.356	1.003(0.815-1.234)	0.580
**Blood loss**		**0.789**		**0.199**
≤200 ml	1 (Ref)		1 (Ref)	
200-400 ml	0.901(0.723-1.122)	0.350	0.900(0.715-1.134)	0.373
>400 ml	1.049(0.816-1.347)	0.710	0.736(0.552-0.980)	0.036
**Lymph node dissection**		**0.004**		**0.045**
D1	1 (Ref)		1 (Ref)	
D2	1.019(0.815-1.275)	0.869	1.024(0.801-1.308)	0.851
D3	0.708(0.560-0.894)	0.004	0.771(0.599-0.992)	0.043

Ref = reference category.

*Derived from tests of HR for prognostic factors in univariate model adjusted for treatment group in Cox proportional-hazards model.

†Cox-regression analysis, controlling for prognostic factors listed in table.

As shown in table 3 and 4, D1 group got significantly more benefit than D2 or D3 group only for Borrmann I and N3 disease, the hazard ratios for death in the D1 group were 0.618 (95% CI, 0.399-0.958; P = 0.031) and 0.369 (95% CI, 0.162-0.841; P = 0.018), respectively.

**Table 3:** Tests for Heterogeneity of Treatment Effect According to the Clinicopathological Characteristics of the D1 and D3 Patients.

**Subgroup**	**D1 surgery**	**D3 surgery**	**HR (95% CI)**	**P**
	
	**No. of deaths/no. of patients**			
**Total**	150/187	137/191	1.407(1.113-1.779)	
**Age (years)**				
≤55	49/66	70/107	1.384(0.955-2.004)	0.086
>55	101/121	67/84	1.242(0.911-1.693)	0.170
**Sex**				
Women	41/51	44/58	1.248(0.815-1.912)	0.309
Men	109/136	93/133	1.463(1.103-1.939)	0.008*
**Tumor size**				
≤5	91/125	74/113	1.401(1.028-1.910)	0.033*
5-7	30/32	42/50	1.412(0.875-2.279)	0.158
>7	29/30	21/28	1.917(1.075-3.419)	0.027*
**Tumour site**				
Upper stomach	40/44	37/42	1.116(0.707-1.762)	0.638
Middle stomach	35/43	29/45	1.641(0.984-2.735)	0.058
Lower stomach	64/88	59/90	1.482(1.038-2.117)	0.030*
Whole stomach	11/12	12/14	1.574(0.685-3.618)	0.286
**Gross appearance**				
Borrmann types I	37/58	35/54	1.100(0.688-1.759)	0.691
Borrmann types II	33/41	32/43	1.781(1.069-2.967)	0.027*
Borrmann types III	66/73	56/77	1.685(1.175-2.416)	0.005*
Borrmann types IV	14/15	14/17	1.708(0.810-3.600)	0.160
**Tumour stage**				
T1	29/41	24/44	1.723(0.992-2.992)	0.054
T2	30/44	29/42	1.282(0.754-2.178)	0.359
T3	79/89	71/92	1.431(1.037-1.977)	0.029*
T4	12/13	13/13	1.071(0.485-2.365)	0.866
**Clinical node status**				
Negative	4/5	2/5	3.070(0.533-17.703)	0.209
Positive	146/182	135/186	1.382(1.091-1.752)	0.007*
**Lymph-node stage**				
N0	50/69	42/69	1.781(1.168-2.718)	0.007*
N1	64/75	60/81	1.504(1.055-2.145)	0.024*
N2	24/30	19/24	1.116(0.609-2.044)	0.723
N3	12/13	16/17	0.369(0.162-0.841)†	0.018*
**Histological type**				
Differentiated	62/99	49/82	1.143(0.761-1.563)	0.231
Undifferentiated	62/99 50/88	49/82 56/109	1.047(0.773-1.732)	0.334
**Joint organ removal**				
None	127/160	104/157	1.703(1.309-2.217)	0.000*
Pancreas or spleen	6/7	14/14	0.754(0.285-1.996)	0.569
Liver or gall	9/11	9/9	0.532(0.200-1.415)	0.206
Transverse colon	8/9	10/11	0.403(0.144-1.133)	0.085
**Gastrectomy**				
Total	20/22	26/29	1.259(0.697-2.274)	0.444
Subtotal	130/165	111/162	1.495(1.156-1.932)	0.002*
**Blood transfusion**				
No	63/87	52/80	1.316(0.909-1.906)	0.146
Yes	87/100	85/111	1.485(1.097-2.011)	0.011*
**Blood loss**				
≤200	41/54	41/49	0.858(0.555-1.328)	0.493
200-400	68/88	56/83	1.623(1.130-2.332)	0.009*
>400	41/45	40/59	2.060(1.327-3.198)	0.001*

The P values are for hazard ratios for death in the group assigned to D1 lymphadenectomy and the group assigned to D3 lymphadenectomy, with 95% confidence intervals.

The surgery is better for D1 lymphadenectomy when the HR is <1; and is better for D3 lymphadenectomy when the HR is >1.

†Significantly better for D1 lymphadenectomy.

*Considered significant.

**Table 4:** Tests for Heterogeneity of Treatment Effect According to the Clinicopathological Characteristics of the D1 and D2 Patients.

**Subgroup**	**D1 surgery**	**D2 surgery**	**HR (95% CI)**	**p**
	
	**No. of deaths/no. of patients**			
**Total**	150/187	157/189	0.982(0.785-1.229)	
**Age (years)**				
≤55	49/66	88/107	0.877(0.618-1.244)	0.462
>55	101/121	69/82	1.016(0.748-1.380)	0.919
**Sex**				
Women	41/51	40/52	1.308(0.843-2.029)	0.231
Men	109/136	117/137	0.878(0.676-1.140)	0.329
**Tumor size**				
≤5	91/125	97/121	0.921(0.692-1.227)	0.575
5-7	30/32	34/40	1.178(0.714-1.941)	0.522
>7	29/30	26/28	1.143(0.672-1.945)	0.622
**Tumour site**				
Upper stomach	40/44	32/39	1.205(0.752-1.930)	0.438
Middle stomach	35/43	41/50	1.086(0.691-1.706)	0.721
Lower stomach	64/88	72/87	0.806(0.575-1.130)	0.212
Whole stomach	11/12	12/13	1.062(0.464-2.429)	0.887
**Gross appearance**				
Borrmann types I	37/58	45/55	0.618(0.399-0.958) †	0.031*
Borrmann types II	33/41	31/44	1.189(0.727-1.945)	0.490
Borrmann types III	66/73	66/74	1.327(0.938-1.878)	0.110
Borrmann types IV	14/15	15/16	1.105(0.528-2.312)	0.791
**Tumour stage**				
T1	29/41	35/40	0.755(0.458-1.243)	0.269
T2	30/44	32/45	1.093(0.661-1.808)	0.729
T3	79/89	75/87	1.073(0.782-1.473)	0.662
T4	12/13	15/17	0.854(0.383-1.906)	0.700
**Clinical node status**				
Negative	4/5	2/3	2.654(0.458-15.362)	0.276
Positive	146/182	155/186	0.965(0.770-1.210)	0.759
**Lymph-node stage**				
N0	50/69	46/60	1.004(0.672-1.500)	0.984
N1	64/75	80/93	1.040(0.748-1.446)	0.814
N2	24/30	21/26	0.820(0.455-1.477)	0.508
N3	12/13	10/10	0.376(0.153-0.922)†	0.033*
**Histological type**				
Differentiated	62/99	55/88	1.033(0.921-1.783)	0.136
Undifferentiated	50/88	56/101	1.157(0.833-1.947)	0.390
**Joint organ removal**				
None	127/160	124/148	0.960(0.749-1.229)	0.745
Pancreas or spleen	6/7	13/16	1.340(0.502-3.575)	0.559
Liver or gall	9/11	4/8	2.959(0.796-10.992)	0.105
Transverse colon	8/9	16/17	0.659(0.277-1.566)	0.345
**Gastrectomy**				
Total	20/22	20/21	1.089(0.583-2.036)	0.788
Subtotal	130/165	137/168	0.970(0.763-1.233)	0.802
**Blood transfusion**				
No	63/87	70/85	0.900(0.640-1.265)	0.543
Yes	87/100	87/104	1.056(0.785-1.422)	0.718
**Blood loss**				
≤200	41/54	57/70	0.920(0.616-1.375)	0.685
200-400	68/88	67/73	0.703(0.501-0.986) †	0.041*
>400	41/45	33/46	1.870(1.175-2.977)	0.008*

The P values are for hazard ratios for death in the group assigned to D1 lymphadenectomy and the group assigned to D2 lymphadenectomy, with 95% confidence intervals.

The surgery is better for D1 lymphadenectomy when the HR is <1; and is better for D2 lymphadenectomy when the HR is >1.

†Significantly better for D1 lymphadenectomy.

*Considered significant.

D3 group has significantly more benefit than D1 and D2 surgery in the subgroups of cases with ≤5 cm and >7 cm tumors, the lower third tumor, Borrmann II and III types, T3 stage, positive clinical node, N0 and N1 disease, no joint organ removal, the subtotal gastrectomy, blood transfusion and 200-400 ml blood loss. There was no evidence indicating that D2 surgery has any significant benefit for these subgroups.

## Discussion

In the study, we found significant improvement in overall survival with D3 surgery compared to D1 surgery. Furthermore, no significant difference was found in the incident rates of major surgery-related complications between the two groups, which was similar to the results in the trials done in Hong Kong,[14] the UK,[5] and Dutch[15].

We conducted a post hoc subgroup analysis including thirteen variables.To be interest, the results indicated that D1 surgery got significantly more benefit than D3 surgery for N3 disease, while D3 surgery has significantly more benefit than D1 surgery in the subgroups of N0 and N1 disease. Since this result was from a post hoc subgroup, it might be a false positive owing to multiple testing,[16] the possible survival benefit of D3 lymphadenectomy in node-negative patients will need to be clarified in further studies.

In a review article, Sun Hu Yang et alreported that there was no difference in the 3- or 5-year survival between D1 and D2[17]. In the study, the 5-year overall survival was 37.4% for D1 group and 48.7% for D3 group, and the period of diagnosis didn't affect survival (p = 0.084, log-rank test)[18]. It can be seen that long-term survival is lower, when comparing our results with historical report, in which the observed 5-year survival rates were 53.6% and 59.5%, respectively[13]. This result indicated that the time of diagnosis of malignant tumours as well as gastric cancer is much later in China than in other countries, especially western countries.

In this study, we found that more than 60% of both the D1 and D3 patients had lymph node metastases which was higher than the report of Bunt et al[19]. In the D3 group, which includes lymph node dissection of the N1, N2 and N3 level, there were 42% being classified as N1, 13% as N2, and 9% as N3. The extended surgery is considered to be related to the risk of operative morbidity and mortality[6]. The mortality for gastrectomy in Western countries was usually5% and even approaches 16% in some trials[7-9].

Robert C G et al reported that the overall 5-year survival rate for the 286 patients undergoing gastrectomy with additional organ resection was 32%, which was significantly less than the gastrectomy-alone group[20]. Besides the surgery extent, the participating surgeons' operative skill and experience, and the workload cases are also important factors for survival rates[21,22]. There are many studies having reported a relationship between the number of cases treated in a hospital and the outcomes of cancer treatment[22-27]. Moreover, the uniformity of treatment is also important. Our study was carried out in a hospital that performs a high volume of nodal dissections for gastric cancer with low morbidity and mortality rates. In our study, all participating surgeons were of the same department, which minimizes the variation in individual operating skill and management, and did an equal number of D1 and D3 resections during the trial, which balances the comparisons between the two groups without bias to individual surgeons' skill. Therefore, the experience as a result of caseload, surgical skill, and the case selection are very important[5,28,29].

## Conclusions

As D3 gastrectomy is associated with low mortality and adequate survival time when performed in selected institutions that have had sufficient experience with the operation and with post-operative management, we recommend D3 lymphadenectomy for patients with curable gastric cancer except for patients with Borrmann I disease who are more suitable for D1 surgery.

## Competing interests

The authors declare that they have no competing interests.

## Authors' contributions

HZ and PL participated in the design of the study. HZ and DW performed the statistical analysis and drafted the manuscript. CGL, YM, RNS, and SBW participated in the coordination of the study. All authors read and approved the final manuscript.

## Pre-publication history

The pre-publication history for this paper can be accessed here:

http://www.biomedcentral.com/1471-2407/10/308/prepub
